# Cardioprotective effects of asiaticoside against diabetic cardiomyopathy: Activation of the AMPK/Nrf2 pathway

**DOI:** 10.1111/jcmm.18055

**Published:** 2023-12-19

**Authors:** Chennian Xu, Lin Xia, Dengyue Xu, Yang Liu, Ping Jin, Mengen Zhai, Yu Mao, Yiwei Wang, Anguo Wen, Jian Yang, Lifang Yang

**Affiliations:** ^1^ Key Laboratory of Gastrointestinal Pharmacology of Chinese Materia Medica of the State Administration of Traditional Chinese Medicine, Department of Pharmacology, School of Pharmacy Air Force Medical University Xi'an Shaanxi China; ^2^ Department of Cardiovascular Surgery, Xijing Hospital Air Force Medical University Xi'an Shaanxi China; ^3^ Department of Cardiovascular Surgery General Hospital of Northern Theatre Command Shenyang Liaoning China; ^4^ School of Biomedical Engineering, Faculty of Medicine Dalian University of Technology Dalian China; ^5^ Department of Cardiothoracic Surgery The 79th Group Military Hospital of the Chinese People's Liberation Army Liaoyang Liaoning Province China; ^6^ Department of Anesthesiology Xi'an Children's Hospital Xi'an Shaanxi China

**Keywords:** AMPK/Nrf2, asiaticoside, autophagy, diabetic cardiomyopathy, oxidative stress

## Abstract

Diabetic cardiomyopathy (DCM) is a chronic microvascular complication of diabetes that is generally defined as ventricular dysfunction occurring in patients with diabetes and unrelated to known causes. Several mechanisms have been proposed to contribute to the occurrence and persistence of DCM, in which oxidative stress and autophagy play a non‐negligible role. Diabetic cardiomyopathy is involved in a variety of physiological and pathological processes. The 5′ adenosine monophosphate‐activated protein kinase/nuclear factor‐erythroid 2‐related factor 2 (AMPK/Nrf2) are expressed in the heart, and studies have shown that asiaticoside (ASI) and activated AMPK/Nrf2 have a protective effect on the myocardium. However, the roles of ASI and AMPK/Nrf2 in DCM are unknown. The intraperitoneal injection of streptozotocin (STZ) and high‐fat feed were used to establish the DCM models in 100 C57/BL mice. Asiaticoside and inhibitors of AMPK/Nrf2 were used for intervention. Cardiac function, oxidative stress, and autophagy were measured in mice. DCM mice displayed increased levels of oxidative stress while autophagy levels declined. In addition, AMPK/Nrf2 was activated in DCM mice with ASI intervention. Further, we discovered that AMPK/Nrf2 inhibition blocked the protective effect of ASI by compound C and treatment with ML‐385. The present study demonstrates that ASI exerts a protective effect against DCM via the potential activation of the AMPK/Nrf2 pathway. Asiaticoside is a potential therapeutic target for DCM.

## 1 INTRODUCTION

Diabetic cardiomyopathy (DCM) is a chronic microvascular complication of diabetes.^1^, ^2^, ^3^ It is defined as ventricular dysfunction occurring in patients with diabetes and unrelated to known causes. It is closely related to the high incidence and mortality of cardiovascular disease in diabetes patients.^4^, ^5^ DCM is a myocardial‐specific disease that increases the risk of heart failure and death in diabetes patients independent of vascular disease.^6^ Its pathogenesis is complex.^7^ Myocardial fibrosis, cardiac systolic and diastolic dysfunction and heart failure are often seen pathophysiologically.^6^, ^7^, ^8^ In recent studies, autophagy has become a hot topic in the study of DCM mechanisms.^9^, ^10^ Studies have shown that mitochondrial structural and functional damage and mitochondrial autophagy are related to diabetic heart disease. Apart from this, autophagy in the hearts of diabetic animal models has been inhibited. The reduction of autophagy may be an adaptive response that can prevent myocardial injury in type I diabetes. However, autophagy may play different roles in myocardial cell injury in animal models of type II diabetes.^11^ At present, the treatment of DCM lacks targeted drugs,^12^ and asiaticoside (ASI) has strong antioxidative stress and antifibrosis effects.^12^ The triterpenoid saponin ASI is a natural compound isolated from *Centella asiatica*. Experimental evidence indicates that it has extensive biological activity in in vitro cell cultures as well as in in vivo animal models.^13^ It has been confirmed that it has antioxidant, anti‐inflammatory, antiviral, wound‐healing promoting and other pharmacological effects.^14^, ^15^ The researchers found that the ASI compound protects against myocardial ischaemia/reperfusion injury adjustment by attenuating oxidative stress and apoptosis by activating the PI3K/AKT/GSK3 β pathway in vivo and in vitro.^16^

In this study, streptozotocin (STZ) combined with a high‐fat diet (HFD) was used to induce the DCM mice model, and ASI was used as an intervention to explore the protective effect of ASI on DCM myocardial injury and the regulation of 5’adenosine monophosphate‐activated protein kinase/nuclear factor‐erythroid 2‐related factor 2 (AMPK/Nrf2) signalling.

## 2 MATERIALS AND METHODS

### 2.1 Animals

The experimental design and scheme of this study adhered to the United States *National Institutes of Health Guidelines on the Use of Laboratory Animals* and were approved by the ethics committee of the Air Force Medical University (No. 20230262, approved 4 March 2023) and conform to NIH Publication N0 8523, revised 1996. The C57BL/6 male mice, weighing 22.18 ± 2.14 g and healthy for 8–10 weeks, were purchased from the Experimental Animal Center of Air Force Medical University. The mice were raised in a 12‐h light/dark environment, at a temperature of 22–25°C and a humidity level of 55%–60%, to ensure that the mice could freely obtain rat food and water.

### 2.2 Reagents

Asiaticoside (C_48_H_78_O_20_, ≥95%), triphenyl tetrazolium chloride, and 4′,6‐diamidino‐2‐phenylindole dihydrochloride were purchased from Sigma‐Aldrich (St. Louis, MO, USA). Enzyme‐linked immunosorbent assay (ELISA) kits for detecting lactate dehydrogenase (LDH) and connective tissue growth factor (CTGF) levels were purchased from the Institute of Nanjing Jiancheng Bioengineering Institute (Nanjing, Jiangsu, China). Primary antibodies against AMPK, phospho‐AMPK, Nrf2, heme oxygenase 1 (HO‐1) and Beclin1, Atg5, LC3B, were purchased from Abcam (Cambridge, MA, USA). Primary antibodies against glyceraldehyde 3‐phosphate dehydrogenase (GAPDH) were purchased from Cell Signalling Technology (Boston, MA, USA). Goat antirabbit and goat antimouse secondary antibodies were purchased from Zhongshan Company (Beijing, China). Dihydroethidium, which detects the generation of reactive oxygen species (ROS) in cardiac tissues, was purchased from Invitrogen (Carlsbad, CA, USA). Compound C (CC) and ML‐385 were purchased from Selleck Chemicals (Houston, TX, USA).

The high‐resolution ultrasound imaging system (D700, Vinno, Suzhou, China) was purchased from Vinno Company, and the FV2000 laser confocal microscope was purchased from Olympus in Japan.

### 2.3 Experimental protocol

All the 100 C57/BL mice were randomly divided into five groups according to the random number table method, with 20 mice in each group: the control group (sham group), the diabetes cardiomyopathy group (DCM group), the ASI [10 mg/(kg day)] intervention group (ASI + DCM group), the DCM group treated with ASI combined with AMPK inhibitor CC (ASI + CC + DCM group), and the DCM group treated with ASI combined with Nrf2 inhibitor ML‐385 (ASI + ML‐385 + DCM group).

The sham group was fed normal diets, and the other groups were fed HFDs until the end of the experiment. After 1 month, except for the normal control group, STZ (soluble in sodium citrate buffer, concentration 1%) was injected in the other groups intraperitoneally after they had fasted for 12 h, with an injection volume of 35 mg/kg. After the injection of STZ, the groups continued to receive high‐fat feed. We changed the padding every day to keep it dry to ensure enough water consumption. Seventy‐two hours after the STZ injection, the fasting blood glucose (FBG) in the tail vein was measured. A fasting blood glucose level >16.7 mmol/L was considered a successful model. Then, the therapeutic drug intervention was performed. The dosage of ASI was 10 mg/(kg day), which was dissolved in a 0.9% sodium chloride solution. The ASI + DCM group was injected intraperitoneally with ASI [10 mg/(kg day)]; the ASI + CC + DCM group was injected intraperitoneally with ASI [10 mg/(kg day)] and CC [0.25 mg/(kg day)]. The sham group and the DCM group were gavaged with a 0.9% sodium chloride solution at the same dose of ASI. At the end of the fifth month of the experiment (the end point), the mental state, water consumption, hair colour and activity of the mice in each group were recorded. On the 1st, 32nd, 42nd, 56th, 84th and 112th days of the experiment, fasting blood glucose levels in the tail vein blood were measured. The deaths of the mice in each group were recorded and calculated. The survival curve was plotted, and the survival of mice in each group was described.

The mice were randomly divided into the following groups: (1) sham group, (2) DCM group, (3) ASI + DCM group, (4) ASI + CC + DCM group, (5) ASI + ML‐385 + DCM group. ASI (10 mg/kg) was injected intraperitoneally. Compound C (0.25 mg/kg) and ML‐385 (30 mg/kg) were injected intraperitoneally. The concentrations of ASI, compound C (CC) and ML‐385 used are based on information from previous studies.^18^, ^19^, ^20^, ^21^ The mice in the sham group were injected intraperitoneally with the same volume of saline every day. All intervention drugs were administered on the 32nd day of the experiment and then every other day until the end of the experiment.

### 2.4 Sequencing data collection

The related data were obtained from the Gene Expression Omnibus database (GSE161052). The mRNA profiles of the myocardial tissue of the C57BL/6 mice were divided into a normal control group (Control) and a diabetic cardiomyopathy model group (DCM). RNA sequencing was performed on the Illumina sequencing platform (HiSeqTM 2500). Further bioinformatics analyses, including co‐expression and functional enrichment analysis of deregulated mRNAs, were performed.

### 2.5 Echocardiogram and hemodynamic measurement

C57BL/6 mice were anaesthetised with 2% isoflurane. Then, the front chests of the mice were depilated and fixed on a constant temperature platform. During the measurement, 2% isoflurane was used to keep the mice anaesthetised. Transthoracic echocardiography was performed using a high‐resolution ultrasound imaging system (D700, Vinno, Suzhou, China). The electrocardiographic electrode of the constant temperature platform was connected to the limbs for electrocardiographic monitoring. Using M‐mode ultrasound, the heart was observed on the short axis at the level of the middle papillary muscle. We collected the images for cardiac function analysis. Parameters such as the ejection fraction and the shortening fraction were measured with Vevo software.

### 2.6 Histopathological examination

The end point of the model establishment experiment was to quickly remove the mouse hearts from the animals in each group and then wash the blood cells with precooled phosphate buffered saline (PBS). The heart was fixed with 4% paraformaldehyde for 48 h and embedded in a paraffin block by the conventional dewaxing method. Histological observations were performed. Haematoxylin–eosin staining and Masson trichromatic staining were used to determine the cross‐sectional area of the myocardial cells and the degree of fibrosis. The cross‐sectional area and fibrosis area were analysed quantitatively with Image J software.

### 2.7 Enzyme‐linked immunosorbent assay

Following the manufacturer's instructions, we used the ELISA kit to evaluate the activity of serum LDH and creatine kinase to evaluate myocardial necrosis. The activity of superoxide dismutase (SOD) and the content of malondialdehyde in the heart tissue was determined using the ELISA kit of Nanjing Jiancheng Institute of Biological Engineering.

### 2.8 Western blotting

Myocardial ventricular tissue was dissolved in RIPA lysate containing 1% protease and phosphatase inhibitor. The protein concentration was determined using the Pierce BCA Protein Assay Kit (Thermo Fisher Scientific, Waltham, MA, USA). After electrophoresis and membrane transfer with sodium dodecyl sulfate‐polyacrylamide gel, the myocardial tissue proteins of each group were transferred to the polyvinylidene fluoride membrane. The membrane was sealed in 5% skimmed milk at room temperature for 2 h. The corresponding target bands were cut and co‐incubated with the following antibodies: phophso‐AMPK (1:1000), AMPK (1:1000), Nrf2 (1:1000), HO‐1 (1:1000), Beclin 1 (1:1000), Atg5 (1:1000), histone H3 (1:1000) and GAPDH (1:5000). Next, the second antibody was combined with horseradish peroxidase at room temperature for 2 h. Then the antigen–antibody complex was detected with the enhanced chemiluminescence reagent and imaged with ChemiDoc XRS. The GAPDH was used as the internal reference. Image Lab software was used to analyse the stripe grey value.

### 2.9 Measurement of reactive oxygen species

At the end of the experiment, the mice were anaesthetised with 2% isoflurane, and their hearts were removed quickly. The hearts were rinsed thoroughly in precooled PBS to remove blood cells, and then frozen sections of the myocardial tissue were made. The measurement of ROS was described previously.^7^ The oxo‐fluorescent dye dihydro ethyl pyridine was used to measure intracellular ROS production in myocardial tissue frozen sections. The fresh‐frozen myocardium was incubated with an oxidized fluorescent dye and examined with an Olympus FV1000 (Olympus, Tokyo, Japan) laser confocal microscope according to the manufacturer's instructions. We used Image Pro Plus software (Media Cybernetics, Rockville, MD, USA) to calculate the intensity of the fluorescence.

### 2.10 Observation by transmission electron microscope

The specimens were prepared in accordance with the requirements of the electron microscope. The left ventricular myocardial tissue was cut to a size of 1 mm^3^, then quickly put into 2.5% glutaraldehyde solution and secured overnight at 4°C. The fixed tissues were sent to the electron microscope room of the Stomatological Hospital of the Northern Theater Command General Hospital, Shenyang, China, to make samples. The changes in the autophagic bodies and mitochondria in the myocardial tissue of each group were observed with a transmission electron microscope, and pictures were taken for preservation.

### 2.11 Immunofluorescence

The left ventricular anterior wall tissue of the mice was selected, embedded in paraffin, sectioned, dewaxed and rehydrated, washed with PBS buffer solution 5 times (3 min/time), treated with Triton‐X100 (2 g/L) for 15 min, washed with PBS solution three times (5 min/time), sealed with goat serum at room temperature for 1.5 h and washed with PBS solution three times (5 min/time). Then, 50 μL of LC3B antibody (1:100) was added to each section; the sections were incubated at 4°C for 15 h and washed with PBS solution five times (3 min/time). Then, 50 μL of fluorescent secondary antibody (1:500) was added to each section in the darkroom and washed with PBS solution five times (3 min/time). Then 50 μL of DAPI solution was added to each slice under dark conditions; the slices were washed with PBS solution five times (3 min/time) and sealed with fluorescent anti‐quenching solution. Finally, the images were collected.

### 2.12 Statistical analysis

All statistical tests were performed using GraphPad Prism software (Version 9.0; San Diego, CA, USA). Data were presented as mean ± standard error of the mean. Statistical analyses were performed using one‐way analysis of variance, followed by Tukey's post‐hoc tests. *p* Values <0.05/0.01 were considered statistically significant.

## 3 RESULTS

### 3.1 Sequencing data collection

**FIGURE 1:**
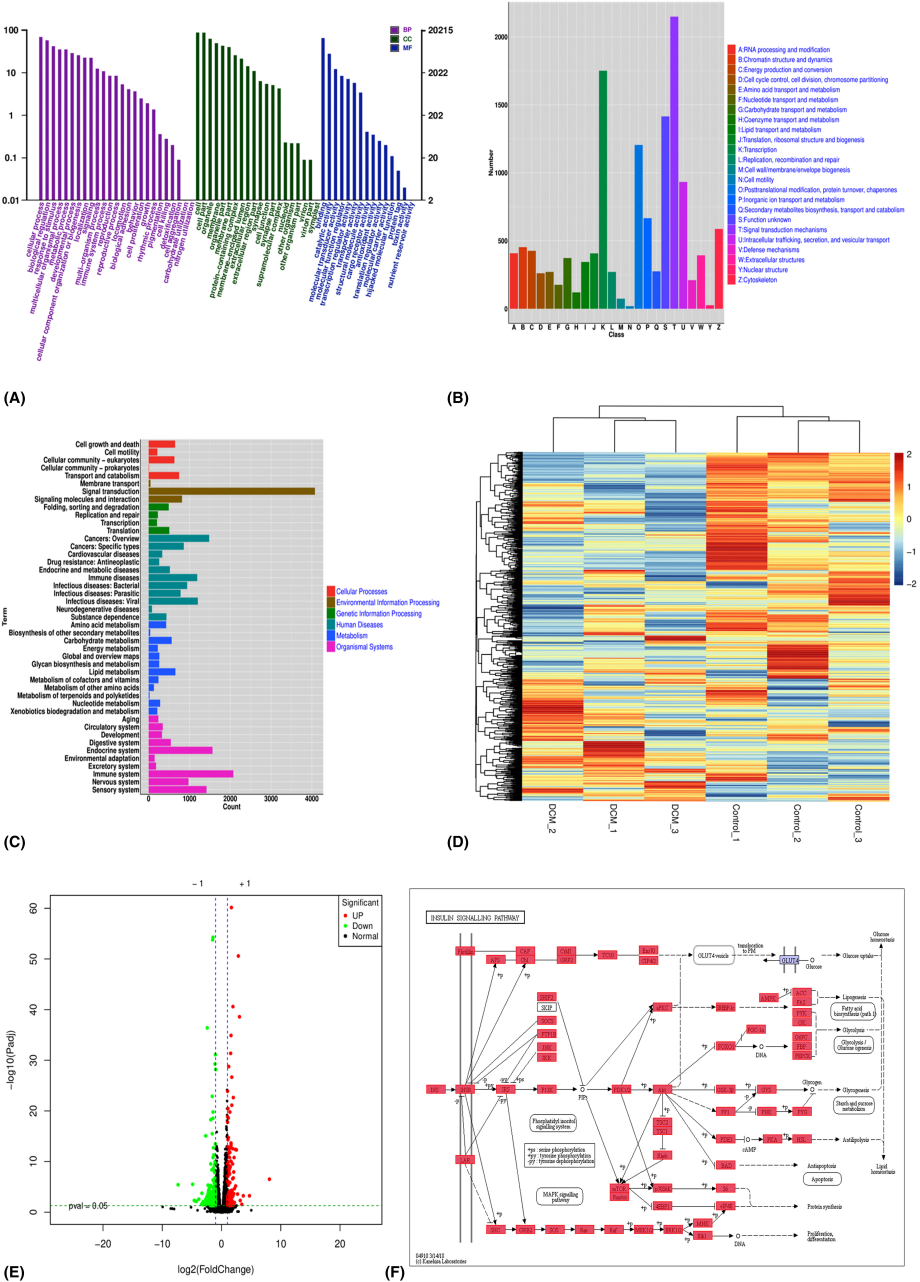
Sequencing data of the diabetic cardiomyopathy model (GSE197999). (A, B) Analysis of module–trait relationships and correlation coefficient of the diabetic cardiomyopathy model based on Gene Expression Omnibus data. (C, D) Enriched Gene Ontology and Kyoto Encyclopedia of Genes and Genomes pathway analyses of mRNAs in the Tan module. The size of the spots corresponds to the number of mRNAs. The colour of the spots represents the *p* value.

To explore the dominant hub genes associated with progression and metastasis, we analysed data from the Gene Expression Omnibus database (GSE161052). All mRNAs were normalized by the μ function via the Limma R package. We applied the WGCNA R software package algorithm to construct a co‐expression network and modules for these six samples. Pearson's correlation matrix was converted into a strengthened adjacency matrix with power *β* = 10. All the selected genes were clustered through a topological overlap matrix‐based dissimilarity measure based on the Dynamic Tree Cut algorithm and divided into 19 clusters labelled with different colours. Then Pearson's correlation coefficient was used to evaluate the interaction of these co‐expression modules. Hierarchical clustering of module eigengenes summarizing the modules was observed in the clustering analysis. Each module labelled with a different colour contained different gene clusters. The red colour represented a positive relationship, whereas the blue colour presented a negative relationship (Figure 1).

### 3.2 Compound C eliminated the improvement of asiaticoside on myocardial tissue structure and cardiac function in diabetes cardiomyopathy

**FIGURE 2:**
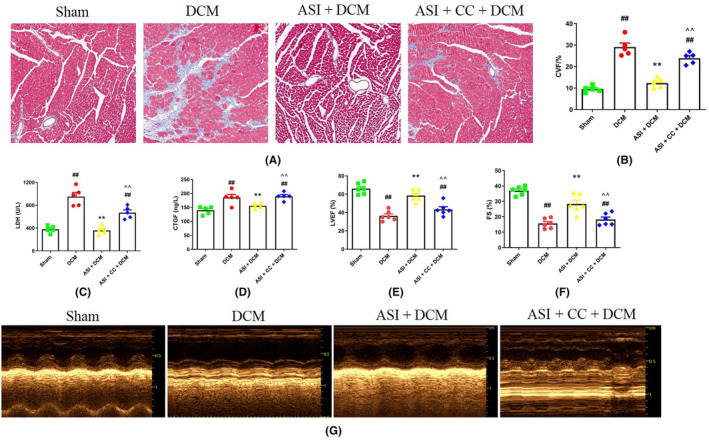
Compound C eliminated the cardioprotective effect of asiaticoside in diabetes cardiomyopathy. (A) Masson staining. (B) Collagen volume fraction. (C) Serum lactate dehydrogenase activity. (D) Serum connective tissue growth factor activity. (E) Left ventricular ejection fraction. (F) Left ventricular fraction shortened. (G) Representative images of echocardiography. The data are expressed as an average ± SEM (*n* = 6). ASI, asiaticoside; CC, compound C; CTGF, connective tissue growth factor; CVF, collagen volume fraction; DCM, diabetes cardiomyopathy; LDH, lactate dehydrogenase; LVEF, left ventricular ejection fraction; LVFS, left ventricular fraction shortened; SEM, standard error of average value. ^##^ *p* < 0.01 compared with the sham group. **p < 0.01 compared with the DCM group. ^^^^ *p* < 0.01 compared with the ASI + DCM group.

To explore the role of ASI in mouse DCM and determine whether the AMPK pathway is involved in the cardiac protection of ASI, the AMPK‐specific inhibitor CC was used in this experiment. Then the changes in cardiac function and myocardial structure were evaluated. As shown in Figure 2A,B, compared with the sham group, ASI treatment significantly improved the level of myocardial fibrosis in the DCM group. In addition, the activities of serum CTGF and LDH in the DCM group were also significantly increased (Figure 2C,D). At the same time, as shown in Figure 2E–G, echocardiographic results showed that the left ventricular ejection fraction (LVEF) and the left ventricular fraction shortened (LVFS), the two main cardiac function indicators in the DCM group, were significantly reduced. As expected, ASI treatment significantly improved cardiac function and structure and reduced myocardial damage (Figure 2A–G). In particular, CC significantly blocked the cardioprotective effect of ASI on myocardial DCM (Figure 2A–G).

### 3.3 Compound C blocked the effect of asiaticoside on antioxidation and improving mitochondrial state in DCM

**FIGURE 3:**
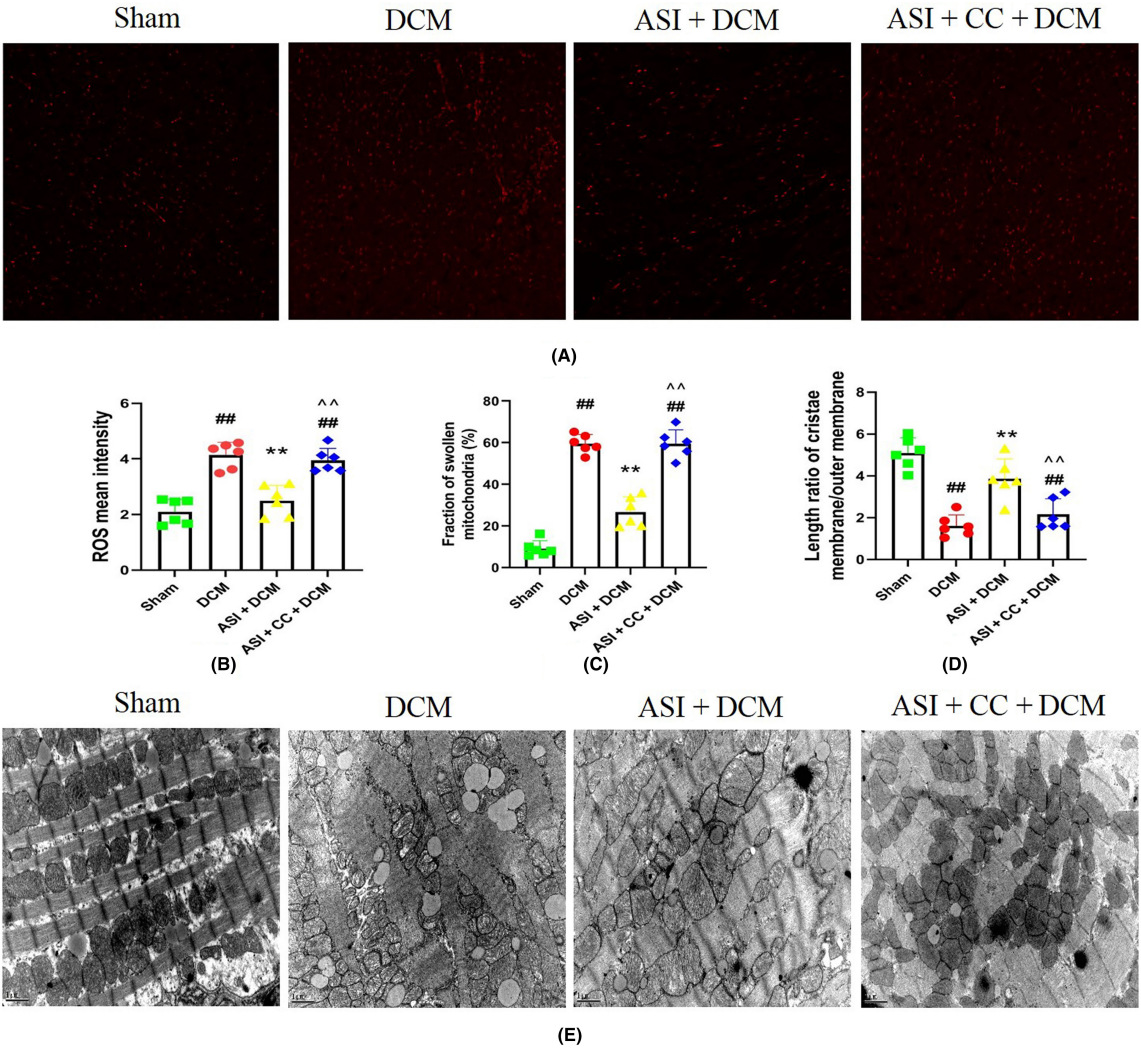
Compound C blocked the antioxidant effect of asiaticoside and improved the mitochondrial state in diabetes cardiomyopathy injury. (A) Representative images of DHE staining in left ventricular tissue (×400). (B) Reactive oxygen species mean intensity. (C) Fraction of swollen mitochondria (%). (D) Length ratio of cristae membrane/outer membrane. (E) Mitochondrial morphology and status (×15,000). The data are expressed as mean ± standard deviation (*n* = 6). ASI, asiaticoside; CC, compound C; DCM, diabetes cardiomyopathy; DHE, dihydroethylpyridine. ^##^ *p* < 0.01 compared with the sham group. **p < 0.01 compared with the DCM group. ^^^^ *p* < 0.01 compared with the ASI + DCM group.

Oxidative stress plays an important role in DCM myocardial injury. As shown in Figure 3A, compared with the sham group, the ROS level in the DCM group also increased significantly (Figure 3B), indicating that the oxidative stress in the DCM group was more severe. Interestingly, compared with the DCM group, ASI treatment can significantly reduce the ROS level, which indicates that ASI reduces oxidative stress in myocardial DCM. However, compound C can reduce the antioxidant effect of ASI in DCM (Figure 3A,B). Transmission microscope observation can directly reflect the changes in the mitochondria. The results of electron microscopy showed that the morphology and structure of the mitochondria in the sham group were normal and that the matrix density was uniform and arranged in an orderly manner. In the DCM group, the mitochondria swelled and deformed, and cristae ruptured and disappeared and were arranged in a disorderly fashion. Compared with the DCM group, the mitochondrial morphology of the ASI + DCM group recovered, the mitochondrial cristae were generally normal and slightly swollen, and the structure was improved, whereas CC could reverse these effects induced by ASI (Figure 3C–E).

### 3.4 Compound C weakened the AMPK activation, the Nrf2 nuclear translocation and the autophagy up‐regulation induced by asiaticoside in diabetes cardiomyopathy injury

**FIGURE 4:**
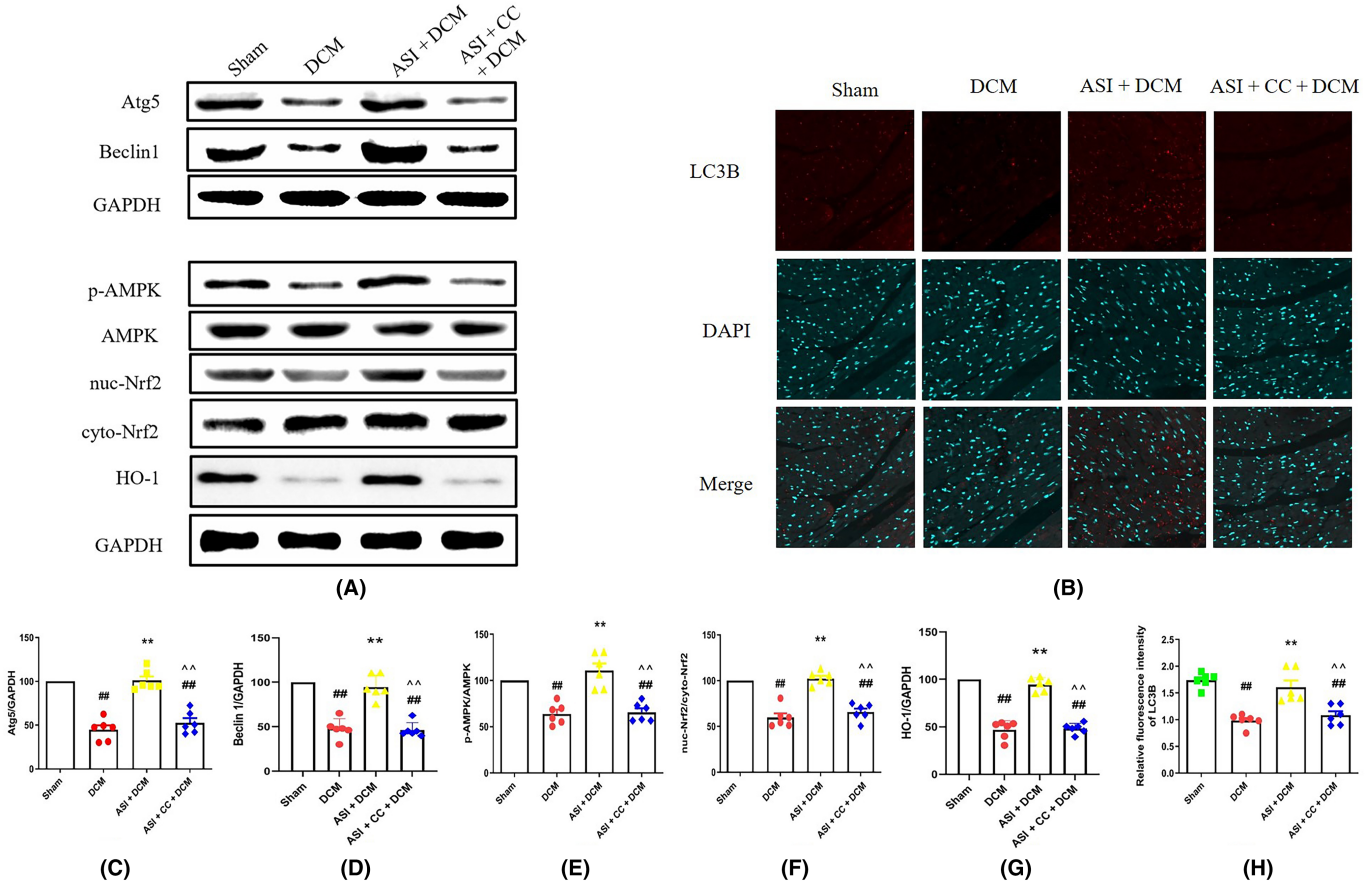
Compound C blocked asiaticoside from activating 5′ adenosine monophosphate‐activated protein kinase/nuclear factor‐erythroid 2‐related factor 2 and up‐regulating autophagy in diabetes cardiomyopathy injury. (A) Atg5, Beclin1, p‐AMPK, AMPK, nuc‐Nrf2, cell‐Nrf2, HO‐1 and GAPDH (internal reference). (B) LC3B immunofluorescence representative image. (C, D) Semi‐quantitative analysis of Atg5 and Beclin1. (E–G) Semi‐quantitative analysis of p‐AMPK, nuc‐Nrf2 and HO‐1. (H) Semi‐quantitative analysis of LC3B immunofluorescence intensity. The data are expressed as mean ± standard deviation (*n* = 6). ASI, asiaticoside; CC, compound C; cyto Nrf2, cytoplasmic Nrf2; DCM, diabetes cardiomyopathy; GAPDH, 3‐phosphate glyceraldehyde dehydrogenase; Nuc‐Nrf2, intranuclear Nrf2; ^##^ *p* < 0.01 compared with the sham group. **p < 0.01 compared with the DCM group. ^^^^ *p* < 0.01 compared with the ASI + DCM group.

The AMPK/Nrf2 pathway is an important mechanism to protect against oxidative stress‐induced injury. To study the molecular mechanism of ASI antioxidant action, we used the Western blot to evaluate the protein levels of Atg5, Beclin1, p‐AMPK, HO‐1, cytoplasmic Nrf2 and nuclear Nrf2. Compared with the sham group, the protein levels of p‐AMPK and nuclear Nrf2 and of Atg5 and Beclin1 decreased (Figure 4). More importantly, ASI treatment can significantly increase the protein levels of p‐AMPK, HO‐1 and nuclear Nrf2 and of Atg5 and Beclin1, which indicates that ASI enhances the activation of the AMPK/Nrf2 pathway in myocardial DCM (Figure 4). However, compound C can reverse the effects induced by ASI (Figure 4).

### 3.5 ML‐385 inhibited the improvement of cardiac oxidative stress, cardiac function and mitochondrial status during asiaticoside‐induced diabetes cardiomyopathy

**FIGURE 5:**
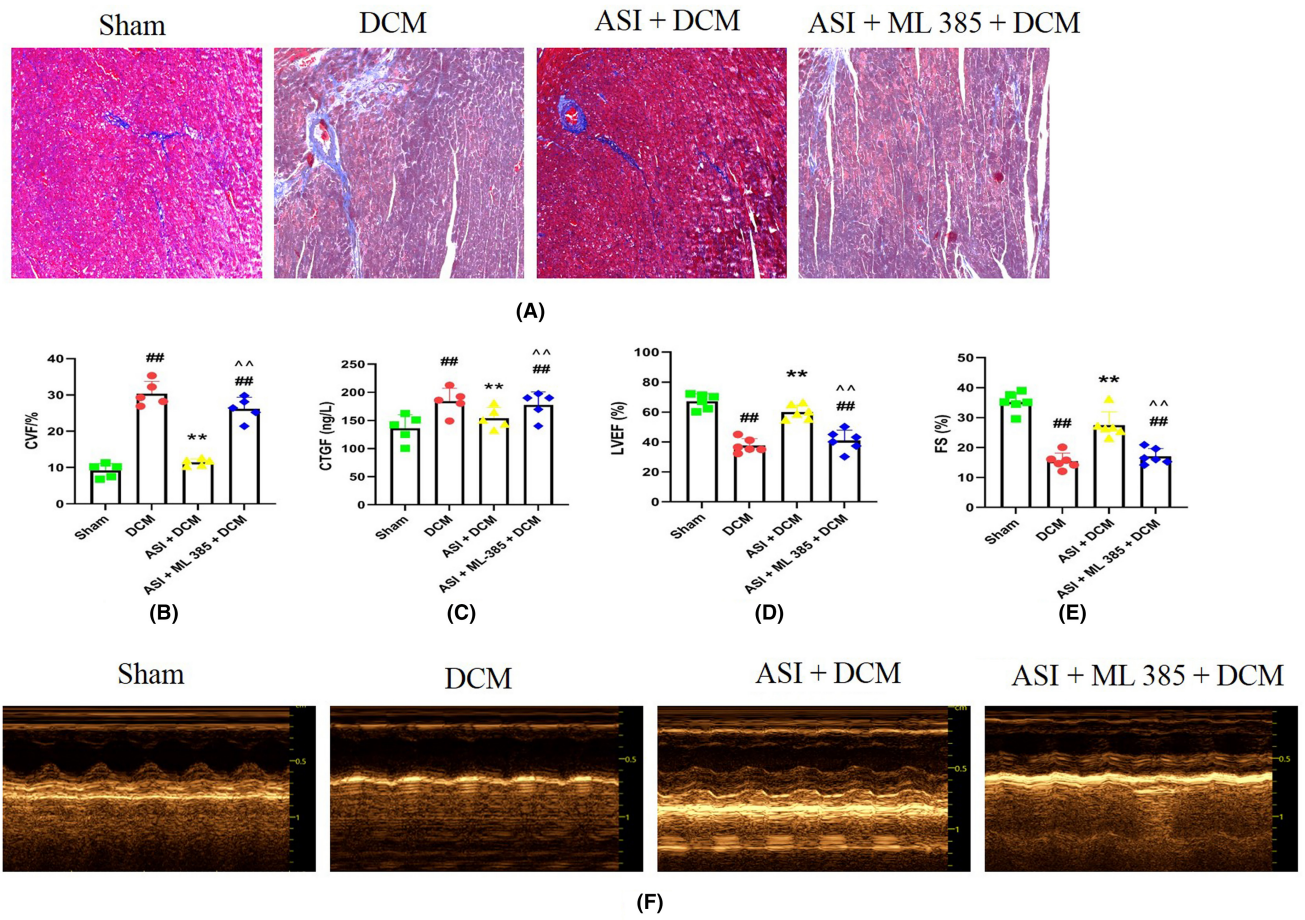
ML‐385 blocked the myocardial protection of asiaticoside in diabetes cardiomyopathy injury. (A) Masson staining. (B) Collagen volume fraction. (C) Serum connective tissue growth factor activity. (D) Left ventricular ejection fraction. (E) Left ventricular fraction shortened. (F) Representative images of echocardiography. The data are expressed as mean ± standard error of the mean (*n* = 6). CVF, collagen volume fraction; ASI, asiaticoside; CTGF, connective tissue growth factor; DCM, diabetes cardiomyopathy; LVEF, left ventricular ejection fraction; LVFS, left ventricular fraction shortened; ML‐385, Nrf2 inhibitor; SEM, standard error of the mean. ^##^ *p* < 0.01 compared with the sham group. **p < 0.01 compared with the diabetic cardiomyopathy group. ^^^^ *p* < 0.01 compared with the ASI + DCM group.

To study whether Nrf2 inhibition could eliminate the cardioprotective effect of ASI in myocardial DCM, ML‐385 was used in these experiments, and then cardiac function and myocardial injury were detected. As shown in Figure 5, myocardial DCM leads to increased myocardial fibrosis and increased serum CTGF activity (Figure 5A–C). In contrast, after myocardial DCM, administration of ASI reduced myocardial fibrosis and serum CTGF activity (Figure 5A–C). In addition, DCM significantly affected cardiac function by reducing LVEF and LVFS (Figure 5D–F). ASI continued to significantly improve cardiac function, and treatment with ML‐385 significantly reversed this situation (Figure 5D–F). However, these protective effects of ASI were also eliminated by treatment with ML‐385, indicating that the cardioprotective effect of ASI in myocardial DCM was related to Nrf2 activity.

**FIGURE 6:**
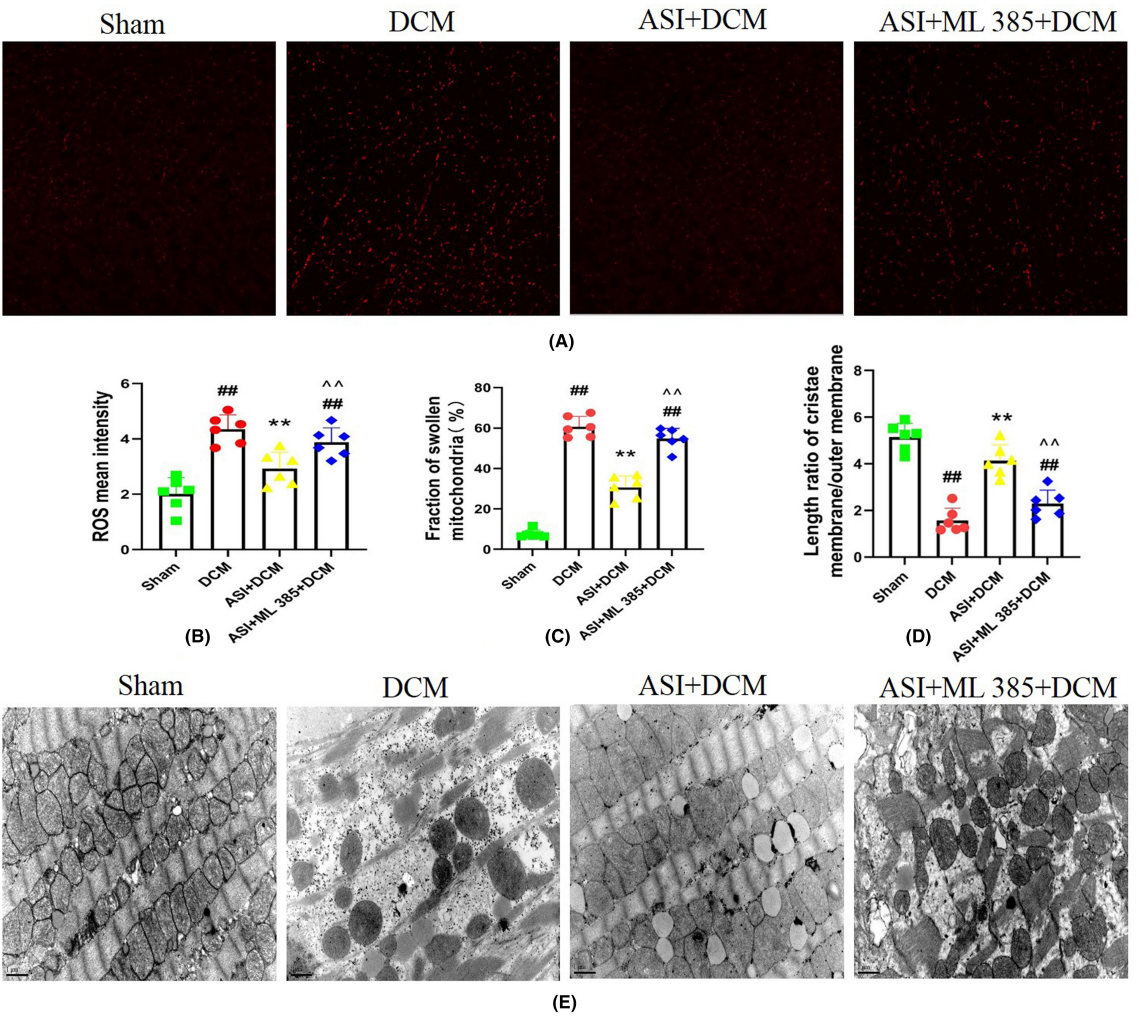
ML‐385 attenuates the antioxidant effect of asiaticoside and the improvement of mitochondrial status in diabetes cardiomyopathy injury. (A) Representative images of dihydroethylpyridine staining in left ventricular tissue (×400). (B) Reactive oxygen species mean intensity. (C) Fraction of swollen mitochondria (%). (D) Length ratio of cristae membrane/outer membrane. (E) Mitochondrial morphology and status (×15,000). The data are expressed as mean ± standard deviation (*n* = 6). ASI, asiaticoside; DCM, diabetes cardiomyopathy; DHE, dihydroethylpyridine; ML‐385, inhibitor of nuclear factor erythroid 2‐related factor 2; ^##^ *p* < 0.01 compared with the sham group. **p < 0.01 compared with the DCM group. ^^^^ *p* < 0.01 compared with the ASI + DCM group.

As shown in Figure 6A, compared with the sham group, the ROS level in the DCM group also increased significantly (Figure 6B), indicating that the oxidative stress in the DCM group was more severe. Interestingly, compared with the DCM group, ASI treatment can significantly reduce the ROS level, which indicates that ASI reduces oxidative stress in myocardial DCM. However, ML‐385 can reduce the antioxidant effect of ASI in DCM (Figure 6A,B). The electron microscopic examination showed that the morphology and structure of mitochondria in the sham group were normal and that the matrix density was uniform and arranged in an orderly manner. In the DCM group, the mitochondria swelled and deformed, the cristae ruptured, disappeared and were arranged in a disorderly fashion. Compared with the DCM group, the mitochondrial morphology of the ASI + DCM group recovered, the mitochondrial cristae were generally normal and slightly swollen, and the structure was improved, whereas ML‐385 could reverse these effects induced by ASI (Figure 6C–E).

### 3.6 ML‐385 attenuated the nuclear translocation of Nrf2 induced by asiaticoside and up‐regulated autophagy but had little effect on the activation of AMPK in diabetes cardiomyopathy injury

**FIGURE 7:**
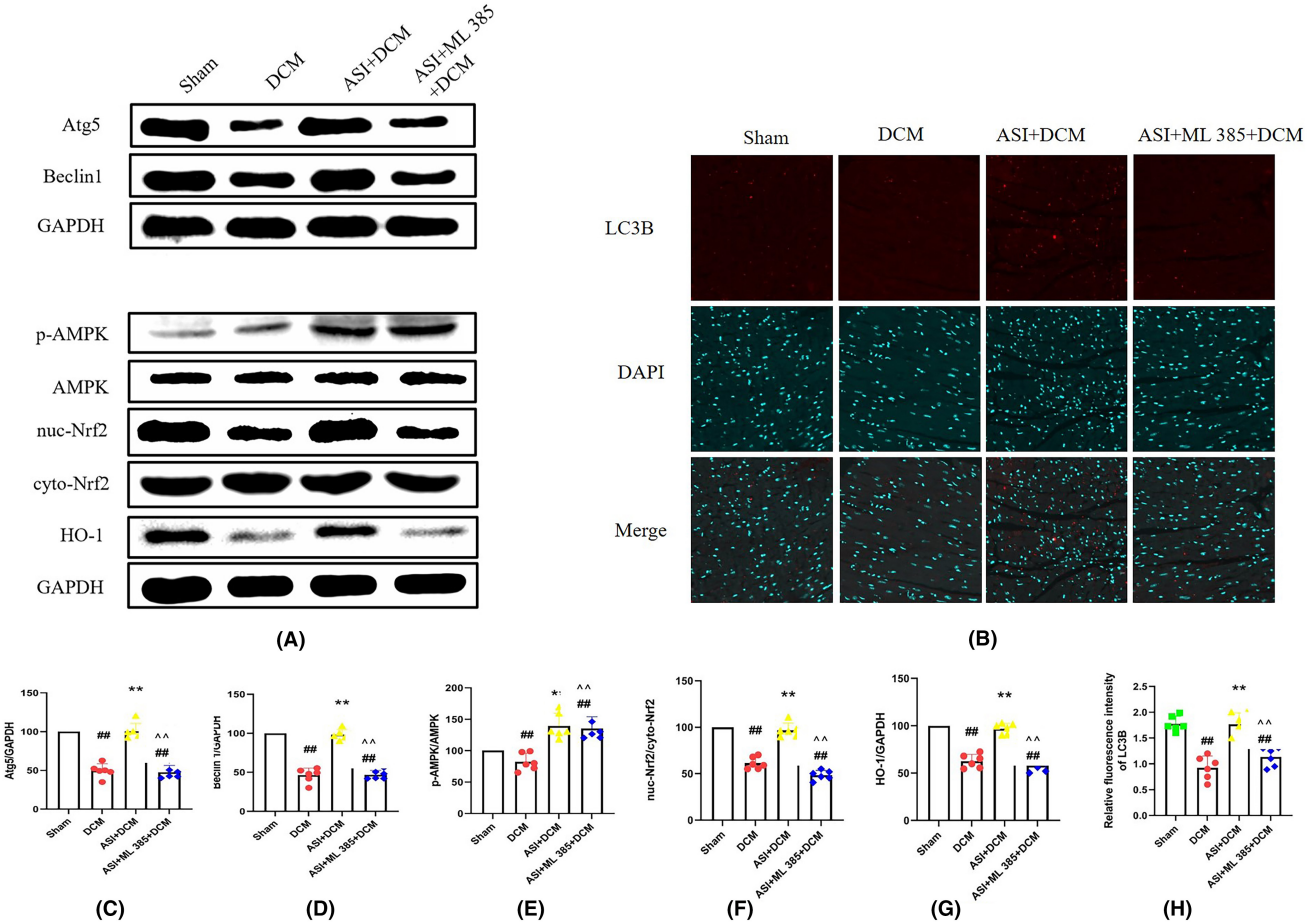
ML‐385 attenuates asiaticoside‐induced nuclear factor erythroid 2‐related factor 2 nuclear translocation but has little effect on the activation of 5′ adenosine monophosphate‐activated protein kinase in myocardial diabetic cardiomyopathy injury. (A) Atg5, Beclin1, p‐AMPK, AMPK, nuc‐Nrf2, cell‐Nrf2, HO‐1 and GAPDH (internal reference). (B) LC3B immunofluorescence representative image. (C, D) Semi‐quantitative analysis of Atg5 and Beclin1. (E–G) Semi‐quantitative analysis of p‐AMPK, nuc‐Nrf2 and HO‐1. (H) Semi‐quantitative analysis of LC3B immunofluorescence intensity. The data are expressed as mean ± standard deviation (*n* = 6). ASI, asiaticoside; cyto Nrf2, cytoplasmic Nrf2; DCM, diabetes cardiomyopathy; GAPDH, 3‐phosphate glyceraldehyde dehydrogenase; ML‐385, Nrf2 inhibitor; Nuc‐Nrf2, intranuclear Nrf2. ^##^ *p* < 0.01 compared with the sham group. **p < 0.01 compared with the DCM group. ^^^^ *p* < 0.01 compared with the ASI + DCM group.

To further explain the correlation between AMPK and Nrf2, we carried out immunoblotting on proteins Atg5, Beclin1, p‐AMPK, intranuclear Nrf2, cytoplasmic Nrf2 and HO‐1. However, it had little effect on the protein levels of p‐AMPK and cytoplasmic Nrf2 (Figure 7). ASI treatment continued to increase the protein levels of Atg5, Beclin1, p‐AMPK, HO‐1 and nuclear Nrf2 (Figure 7). However, ML‐385 can reverse ASI‐induced Nrf2 nuclear translocation but has little effect on the protein levels of p‐AMPK and p‐ACC (Figure 7). These data suggest that AMPK plays an upstream role as an Nrf2 signal in ASI‐mediated cardiac protection against myocardial DCM.

## 4 DISCUSSION

Diabetes cardiomyopathy is a chronic microvascular complication of diabetes.^2^, ^3^, ^10^ It is generally defined as ventricular dysfunction occurring in patients with diabetes and unrelated to known causes. It is closely related to the high incidence and mortality of cardiovascular disease in patients with diabetes.^4^, ^5^ Diabetes cardiomyopathy is a myocardial‐specific disease that increases the risk of heart failure and death in patients with diabetes independent of vascular disease.^6^ Its pathogenesis is complex.^7^ Myocardial fibrosis, cardiac systolic and diastolic dysfunction and heart failure are often seen pathophysiologically.^6^, ^7^, ^8^ Recent studies have focused attention on autophagy as a mechanism of DCM.^10^, ^16^ Studies have shown that mitochondrial structural damage, mitochondrial dysfunction, and mitochondrial autophagy are associated with diabetic heart disease. In addition, autophagy in animal models of diabetes is inhibited.

The reduction of autophagy may be an adaptive response that can prevent myocardial injury in type I diabetes. However, autophagy may play different roles in myocardial cell injury in animal models of type II diabetes.^11^

At present, the treatment of DCM lacks targeted drugs,^4^, ^17^, ^18^ and ASI has strong antioxidative stress and antifibrosis effects. Recently, a variety of natural products/phytochemicals, such as resveratrol, berberine, curcumin, and ASI, have been proven to regulate the autophagy of cardiac myocytes in different ways.^19^, ^20^

The ability of new therapeutic drugs to modify autophagy has gradually become a new therapeutic target. More and more research shows that changes in autophagy are associated with myocardial injury in diabetes.^21^ Researchers have been studying whether autophagy changes aggravate cellular injury or promote cellular survival. For example, overexpression of the antioxidant enzyme heme oxygenase‐1 can prevent cardiac dysfunction in mice with type I diabetes caused by autophagy.^22^ The role of autophagy in type I diabetes‐associated cardiomyopathy remains controversial. The heart damage caused by type I diabetes was weakened in Beclin 1‐deficient mice. The overexpression of Beclin 1 led to increased damage, indicating the role of autophagy in the hearts of mice with type I diabetes.^23^ The role of cardiac autophagy in most HFD‐induced type II diabetes models appears to be adaptive, meaning that enhanced autophagy could have a protective effect on cardiomyopathy in type II diabetes.^22^ In HFD‐treated mice after drug suppression and gene knockout, mTORC1 resumed regulating autophagy and alleviated myocardial infarction in animal models of diabetes.^24^ Similarly, AMPK‐induced dissociation of Bcl‐2: Beclin 1 complex also activates autophagy, which plays a protective role in DCM. Although studies have shown that autophagy is enhanced in patients with type I diabetes, autophagy is inhibited in patients with insulin resistance type II diabetes. Due to the complexity of diabetes, DCM may not be the case and may involve different mechanisms of action.^25^, ^26^ For example, in different types of transgenic animal models of type II diabetes, Akt2 ^−/−^ insulin‐resistant mice and Goto‐Kakizaki rats showed decreased autophagy and increased autophagy, respectively. In H9C2 cells treated with high glucose, insulin resistance induced increased expression of FoxO1 and increased autophagy.^27^ Studies on doxorubicin (DOX)‐induced myocardial cytotoxicity show that DOX enhances autophagy by inhibiting mTOR, up‐regulating Beclin 1 and Atg5, and inhibiting autophagy formation through 3‐MA regulation of PI3K pathway.^28^ In addition, DOX can destroy autophagic flow, cause mitochondrial dysfunction, and increase oxidative stress, which leads to autophagic accumulation and increased cytotoxicity.^29^ Doxorubicin can block the autophagy flux of cardiac myocytes by reducing Beclin 1, inhibiting lysosomal acidification, and slowing down autophagy initiation, thus regulating autophagy flow and improving DOX‐induced cardiotoxicity.

Oxidative stress is considered the key pathogenesis of myocardial DCM injury.^30^, ^31^ The destruction of oxidant‐antioxidant homeostasis, such as ROS production and inhibition of SOD activity, is the main factor leading to oxidative stress in cells. Therefore, clarifying the molecular mechanism of inhibiting oxidative stress may help to prevent myocardial DCM injury. At present, researchers have found that mitochondrial structural damage and function reduction are the main mechanisms of myocardial DCM damage.^32^ The role of mitochondrial regulation and protection in myocardial DCM injury has attracted more and more attention.^33^ Many problems remain to be solved regarding the protective effect of mitochondrial damage and oxidative stress in the process of myocardial DCM injury. For example, proper mitochondrial autophagy can remove damaged mitochondria in time to avoid the release of oxygen‐free radicals from damaged mitochondria and aggravate cell damage.^34^, ^35^ PINK1 can rapidly accumulate on the outer membrane surface of damaged mitochondria. On the one hand, it can promote the phagocytosis of damaged mitochondria by autophagic bodies by recruiting autophagic receptors such as p62 and combining them with LC3B. On the other hand, mitochondrial autophagy can be induced by directly activating parkin and mediating mitochondrial ubiquitin chain synthesis.^36^ A serine/threonine kinase, AMPK, has been identified as the key energy sensor and regulator of cell metabolism; it plays an important role in regulating energy homeostasis and reducing ROS production under normal and ischemic conditions.^37^, ^38^ Due to the change in the intracellular AMP/ATP ratio, AMPK activation occurs during cell energy stress, including DCM.^39^ Therefore, AMPK activation can improve the pathogenesis of metabolic disorders by regulating the expression and activation of various downstream molecules.^40^ In addition to regulating metabolism, AMPK also participates in the regulation of many other cellular processes, including autophagy, endoplasmic reticulum stress, inflammation, and oxidative stress.^40^ Nuclear factor‐erythrocyte 2 related factor 2 is an important transcription factor that can be translocated to the nucleus and can control the expression of many target genes.^41^ It is well known that Nrf2 plays a crucial role in balancing oxidants and antioxidants.

Asiaticoside has good antioxidant and anti‐inflammatory effects, and cardiovascular protection has a variety of protective effects: ASI can inhibit endothelial proliferation, expand blood vessels and improve myocardial ischemia/reperfusion injury, diabetes cardiomyopathy, and heart failure.^41^ However, the mechanism of ASI regulating Nrf2 has not been clarified. Therefore, the purpose of this study was to determine the protective effect of ASI on myocardial DCM injury and determine whether AMPK/Nrf2 signalling is related to ASI‐induced cardiac protection and mitochondrial regulation and protection.

In this study, we demonstrated that ASI could reduce myocardial DCM damage by improving cardiac function and reducing autophagic cell death. These findings are consistent with the results of previous studies. In addition, the cardioprotective effect of ASI on myocardial DCM injury is related to the inhibition of autophagy and oxidative stress. More importantly, we discovered that the AMPK‐specific inhibitor CC blocks AMPK activation and Nrf2 nuclear translocation. It inhibits the antiautophagy and antioxidation effects of ASI, which showed that AMPK/Nrf2 signal activation was essential for ASI to reduce myocardial DCM damage. In addition, ASI protects myocardial DCM injury by activating AMPK/Nrf2, which further proves that the specific Nrf2 inhibitor ML‐385 eliminates Nrf2 nuclear translocation, thereby regulating autophagy, antioxidation, and ASI‐induced cardiac protection but has little effect on AMPK activation. These results suggest that ASI can inhibit autophagy and oxidative stress by activating the AMPK/Nrf2 pathway, thus reducing myocardial damage in DCM.

Autophagy is a kind of mechanism that can degrade misfolded proteins and damaged organelles and is essential for the body to maintain the stability of the intracellular environment.^42^ As a highly conservative housekeeping mechanism, autophagy plays a key role in maintaining energy balance and cell survival to cope with nutrition/energy stress. Destruction of the autophagy of cardiomyocytes, especially the damage of autophagy flux, plays an important role in the pathogenesis of heart failure, hypertrophic cardiomyopathy, dilated cardiomyopathy, cardiac ageing, diabetes cardiomyopathy, and myocardial ischemia/reperfusion injury.^43^ Autophagy is very important for maintaining cardiac function and myocardial homeostasis under basic and stressful conditions. Autophagy has become an important target for preventing heart disease. About two‐thirds of diabetes patients die of heart injury or stroke, so it is important to understand and treat cardiovascular complications caused by diabetes, including DCM.^43^ Autophagy, namely programmed cell death, causes cell death during myocardial DCM injury. The results of a previous study showed that autophagy was the main form of myocardial cell death.^44^ Consistent with the results of other previous studies, we found that ASI alleviated DCM‐induced cardiac dysfunction and myocardial injury, which can be demonstrated by increasing LVEF and LVFS and serum LDH and CTGF activities. At the same time, ASI reduced the death of autophagic cells, which could be proved by significantly reducing the autophagic index. However, the AMPK‐specific inhibitor CC and specific Nrf2 inhibitor ML‐385 can significantly inhibit ASI‐induced cardiac protective changes. These results suggest that AMPK and Nrf2 may participate in cardiac protection and autophagy of ASI in myocardial DCM injury.

Oxidative stress is a prominent pathological feature and plays an important role in the pathogenesis of myocardial DCM injury.^33^ The destruction of oxidant‐antioxidant homeostasis is the main factor leading to cell oxidative stress. Antioxidants such as SOD and glutathione peroxidase protect myocardial cells from oxidative damage. In addition, ASI is a well‐known oxidation inhibitor and plays an antioxidant role in cardiovascular, metabolic, and neurodegenerative diseases.^15^ In this regard, we believe that the cardioprotective effect of ASI is related to the reduction of oxidative stress and mitochondrial damage in myocardial DCM. In this study, the results show that, compared with the sham group, the levels of ROS in the DCM group increase significantly while the autophagic activity decreases. At the same time, compared with the sham group, in the DCM group, the morphology and function of the mitochondria are damaged. As expected, ASI processing DCM reduces the ROS level. However, CC and ML‐385 can block the antioxidant effects induced by ASI in myocardial DCM injury, which indicates that the antioxidant effect of ASI and mitochondrial morphology and function damage are related to AMPK and Nrf2.

**FIGURE 8:**
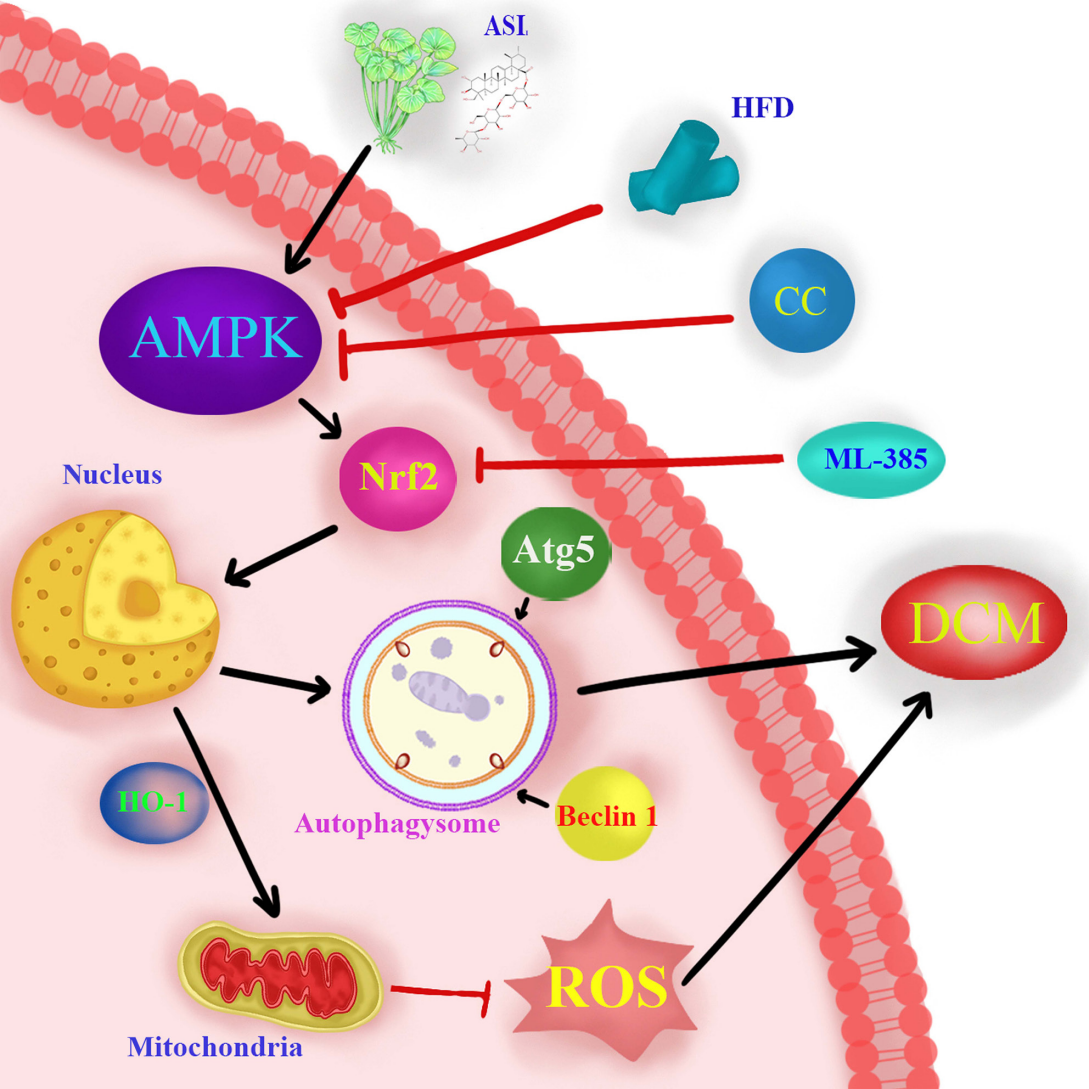
Schematic illustration of asiaticoside‐enhanced 5′ adenosine monophosphate‐activated protein kinase/nuclear factor‐erythroid 2‐related factor 2 axis in diabetic cardiomyopathy. ASI, asiaticoside; CC, compound C; DCM, diabetes cardiomyopathy; HFD, high‐fat diet; ML‐385, Nrf2 inhibitor.

5′ Adenosine monophosphate‐activated protein kinase is the main energy‐sensing serine/threonine kinase that functions as a cardiac energy sensor and maintains energy homeostasis under physiological and pathological conditions.^45^ It is activated in response to cell metabolic stress and then phosphorylated and inactivated to enhance the activity of carnitine palmitoyl transferase 1, thus promoting the oxidation of fatty acids.^46^ In addition, it is increasingly recognized that AMPK is related to circulating hormones and local autocrine and paracrine factors.^47^ In this study, we used the myocardial DCM model to prove that practical treatment with ASI can regulate AMPK and Nrf2. Our results showed that the treatment of DCM with ASI significantly increased the phosphorylation of AMPK, and increased the levels of Nrf2 and HO‐1 in the nucleus. However, CC can block the ASI‐dependent regulation of AMPK and Nrf2 in myocardial DCM, which supports the concept that ASI can regulate myocardial DCM injury through the AMPK/Nrf2 signal pathway. Interestingly, ML‐385 can also block ASI's dependent regulation of Nrf2 but has little effect on AMPK activation, which indicates that Nrf2 acts as the downstream activator of the AMPK signal in the cardiac protection of myocardial DCM injury induced by ASI. Nuclear factor‐erythroid 2‐related factor 2 is a widely expressed transcription factor that regulates the transcription of a series of target genes in response to oxidative stress under basic and pathological conditions.^48^ It has previously been shown that Nrf2 plays a key role in the cardiovascular system by regulating the antioxidant enzyme HO‐1.^49^ According to studies in rodents exposed to DCM and pressure overload, activating Nrf2 through specific interventions can reduce the myocardial infarction area and myocardial hypertrophy, respectively.^50^, ^51^ We found that the levels of Nrf2 and HO‐1 in the nucleus of the DCM group decreased significantly. More importantly, ASI treatment significantly increased the levels of Nrf2 and HO‐1 in the nucleus. It can be predicted that both CC and ML‐385 can inhibit ASI‐induced nuclear Nrf2 and HO‐1 levels. These results further confirmed that the AMPK/Nrf2 signal pathway is involved in the cardiac protection of ASI in myocardial DCM injury (Figure 8).

## 5 CONCLUSION

Our research results suggest that ASI can alleviate DCM myocardial injury by improving mitochondrial status, enhancing autophagy, and reducing oxidative stress. In addition, our results indicate that the AMPK/Nrf2 signalling pathway plays a critical role in regulating the cardioprotective effect of ASI on myocardial DCM injuries and improving mitochondrial morphology. Therefore, we conclude that ASI is a promising intervention for patients with diabetes cardiomyopathy.

## AUTHOR CONTRIBUTIONS

**Chennian Xu:** Conceptualization (equal); data curation (equal); formal analysis (equal); writing – original draft (equal). **Lin Xia:** Formal analysis (equal); investigation (equal). **Dengyue Xu:** Conceptualization (equal); data curation (equal); formal analysis (equal). **Yang Liu:** Conceptualization (equal); investigation (equal); supervision (equal). **Ping Jin:** Supervision (equal); validation (equal). **Mengen Zhai:** Validation (equal). **Yu Mao:** Validation (equal). **Yiwei Wang:** Validation (equal). **Anguo Wen:** Validation (equal). **Jian Yang:** Funding acquisition (equal); project administration (equal); supervision (equal); validation (equal); writing – review and editing (equal). **Lifang Yang:** Funding acquisition (equal); project administration (equal); supervision (equal); validation (equal); writing – review and editing (equal).

## FUNDING INFORMATION

This study was supported by funding from the National Natural Science Foundation of China (82174493, 81774415, 82100513); the General Program of China Postdoctoral Science Foundation (2022M713857); the National Key Research and Development Program of China (2020YFC2008100); the Shaanxi Provincial Fund for Distinguished Young Scholars (2021JC‐49); and the Shaanxi Provincial Key Research and Development Plan (2023‐YBSF‐105).

## CONFLICT OF INTEREST STATEMENT

The authors have no conflicts of interest to declare.

## CONTRIBUTION TO THE FIELD

Diabetes cardiomyopathy (DCM) is a chronic microvascular complication of diabetes, which is generally defined as ventricular dysfunction occurring in patients with diabetes and unrelated to any known causes. Oxidative stress and autophagy play a non‐negligible role in the mechanisms of DCM. The enzyme AMPK and the nuclear factor Nrf2 are expressed in the heart, and studies have shown that administering asiaticoside and activating AMPK/Nrf2 have protective effects on the myocardium. However, the roles of asiaticoside and AMPK/Nrf2 in DCM are unknown. Our study demonstrates that asiaticoside exerts a protective effect against DCM via a potential activation of the AMPK/Nrf2 pathway. Asiaticoside is a potential therapeutic target for DCM.

## Data Availability

The data used in this study are available from the corresponding author upon request.
